# A Comprehensive Systematic Review and Meta-Analysis of the Association between the Neutrophil-to-Lymphocyte Ratio and Adverse Outcomes in Patients with Acute Exacerbation of Chronic Obstructive Pulmonary Disease

**DOI:** 10.3390/jcm11123365

**Published:** 2022-06-11

**Authors:** Angelo Zinellu, Elisabetta Zinellu, Maria Carmina Pau, Ciriaco Carru, Pietro Pirina, Alessandro G. Fois, Arduino A. Mangoni

**Affiliations:** 1 Department of Biomedical Sciences, University of Sassari, 07100 Sassari, Italy; azinellu@uniss.it (A.Z.); carru@uniss.it (C.C.); 2 Clinical and Interventional Pneumology, University Hospital of Sassari (AOU), 07100 Sassari, Italy; elisabetta.zinellu@aousassari.it (E.Z.); pirina@uniss.it (P.P.); agfois@uniss.it (A.G.F.); 3 Department of Medical, Surgical and Experimental Sciences, University of Sassari, 07100 Sassari, Italy; mcpau@uniss.it; 4 Quality Control Unit, University Hospital of Sassari (AOU), 07100 Sassari, Italy; 5 Discipline of Clinical Pharmacology, College of Medicine and Public Health, Flinders University, Bedford Park, SA 5042, Australia; 6 Department of Clinical Pharmacology, Flinders Medical Centre, Southern Adelaide Local Health Network, Bedford Park, SA 5042, Australia

**Keywords:** neutrophil-to-lymphocyte ratio, acute exacerbation of chronic obstructive pulmonary disease, adverse outcomes, mortality, prognostic capacity, biomarker, inflammation

## Abstract

The neutrophil-to-lymphocyte ratio (NLR) predicts adverse outcomes in stable chronic obstructive pulmonary disease (COPD); however, its prognostic role in acute exacerbations (AECOPD) is less clear. We conducted a systematic review and meta-analysis of the association between the NLR on admission and adverse outcomes (mortality, need for mechanical ventilation, transfer to the intensive care unit, length of stay, pulmonary hypertension, or their combination) in AECOPD by searching PubMed, Web of Science, and Scopus from inception to April 2022. Risk of bias and certainty of evidence were assessed using the Joanna Briggs Institute Critical Appraisal Checklist and the Grades of Recommendation, Assessment, Development, and Evaluation, respectively. In 15 studies (n = 10,038 patients), the NLR was significantly associated with the risk of adverse outcomes (odds ratio = 1.054, 95% CI 1.016 to 1.093, *p* = 0.005; low certainty of evidence; standard mean difference = 0.82, 95% CI 0.57 to 1.06, *p* < 0.001; high certainty of evidence). Pooled sensitivity, specificity, and area under the curve were 0.71 (95% CI 0.64 to 0.77), 0.73 (95% CI 0.65 to 0.80), and 0.78 (95% CI 0.74 to 0.81), respectively. In our study, the NLR on admission was significantly associated with adverse outcomes in AECOPD patients, suggesting the potential utility of this biomarker for early risk stratification and management in this group.

## 1. Introduction

Chronic obstructive pulmonary disease (COPD) represents one of the leading causes of death worldwide. Current global COPD-related mortality figures, three million deaths annually, are estimated to increase to over five million deaths annually by 2060 [1,2,3]. Patients with COPD have an increased risk of suffering from other disabling conditions, particularly lung cancer, cardiovascular disease, sarcopenia, anxiety, depression, and cognitive impairment [4,5,6,7,8]. Another common complication is represented by acute exacerbation of COPD (AECOPD), defined by the Global Initiative for Chronic Obstructive Lung Disease (GOLD) as an acute event characterized by a worsening of respiratory symptoms that is beyond normal day-to-day variations and leads to medication changes [9]. Clinically, AECOPD is characterized by the presence of one or more of increased cough, increased dyspnoea, and increased sputum volume and/or change in character [9]. Importantly, AECOPD represents the leading cause of hospitalization and death in COPD patients, with in-hospital, three-month, six-month, and two-year mortality of 6.7%, 18%, 26%, and 31%, respectively [10,11,12].

The in-hospital management of AECOPD includes the administration of antibiotics, bronchodilators, and steroids [9]. However, the wide range of causative factors (i.e., bacterial infections, viral infections, and environmental pollution) suggests that this patient group is heterogeneous and may benefit from more personalized treatment strategies. Therefore, there is an increasing focus on identifying novel prognostic biomarkers that, in combination with clinical assessment, enhance early risk stratification and rapid introduction of individualized care pathways. It has been suggested that an ideal biomarker of AECOPD should reflect the acute increase in airway inflammation commonly observed in this patient group, predict adverse outcomes, and be robustly measured using inexpensive methods [13,14,15]. In this context, combined indexes of inflammation derived from routine haematological parameters, particularly the neutrophil-to-lymphocyte ratio (NLR), are increasingly being recognized as reliable indicators of systemic inflammation and independent predictors of outcomes in stable COPD [16,17]. However, the prognostic role of the NLR specifically in patients with AECOPD has not been investigated until recently.

We sought to address this issue by conducting a systematic review and meta-analysis of studies reporting independent associations, through multivariate analysis, between the NLR on hospital admission and clinical adverse outcomes in patients with AECOPD and assessing the prognostic performance of this biomarker.

## 2. Materials and Methods

### 2.1. Literature Search

We conducted a systematic literature search for articles published in PubMed, Web of Science, and Scopus, between inception and the 15th of April 2022, using the following terms (and their combination): “NLR” or “neutrophil-to-lymphocyte ratio” or “neutrophil lymphocyte ratio” and “AECOPD” or “acute exacerbation of COPD” or “acute exacerbation of chronic obstructive pulmonary disease”. We also hand-searched the reference lists of individual articles to identify additional studies. The inclusion criteria were: (a) full text available, (b) English language used, (c) articles reporting associations between the NLR and adverse outcomes (mortality, length of stay, transfer to the intensive care unit, ICU, need for mechanical ventilation, pulmonary hypertension, or their combination) in patients with AECOPD, (d) and reported odds ratio (OR) with 95% confidence intervals (CI) for adverse outcomes using logistic multivariate analysis. Abstracts and, if relevant, full articles were independently reviewed by two investigators, with a third involved in case of disagreement.

Data extracted included the country where the study was conducted, the year of publication, the age and sex of participants, the sample size, the clinical endpoint studied, the area under the receiver operating characteristic curve (AUROC) with 95% confidence intervals (CIs), and the cut-off values used for the NLR. True positive (TP), false positive (FP), false negative (FN), and true negative (TN) values were either extracted or calculated, by generating 2 × 2 tables, from each study. Sensitivity and specificity were derived using the following formulas: Sensitivity = TP/(TP + FN); Specificity = TN/(FP + TN).

We assessed the risk of bias using the Joanna Briggs Institute (JBI) Critical Appraisal Checklist for case–control studies, with scores  ≥5, 4, and  < 4 indicating low, moderate and high risk, respectively [18], and the certainty of evidence using the Grades of Recommendation, Assessment, Development, and Evaluation (GRADE) Working Group system [19]. The study was conducted in accordance with the PRISMA 2020 statement on the reporting of systematic reviews and meta-analyses (Supplementary Tables S1 and S2) [20]. The protocol was registered in the International Prospective Register of Systematic Reviews (PROSPERO, CRD333137).

### 2.2. Statistical Analysis

Data regarding the associations between the NLR and adverse outcomes, expressed as ORs adjusted for confounding variables and 95% CIs, were extracted. ORs were then transformed into log ORs, and the standard error was calculated based on the corresponding 95% CI. In addition, forest plots of continuous variables, generated from standardized mean differences (SMDs), were used to assess differences in NLR values between patients with and without adverse outcomes (*p* < 0.05 for statistical significance). We assessed heterogeneity using the Q statistic (*p* < 0.10 for statistical significance). I^2^ values <30% and ≥30% indicated no/slight and moderate/substantial heterogeneity, respectively [21]. A random-effect model based on the inverse-variance method was used in the presence of moderate or substantial heterogeneity [21]. Sensitivity analyses were conducted to investigate the effect of sequentially removing individual studies on the overall risk estimate [22]. To evaluate the presence of publication bias, the associations between study size and the magnitude of effect were analysed using the Begg’s adjusted rank correlation test and the Egger’s regression asymmetry test (*p* < 0.05 for statistical significance) [23,24], and the Duval and Tweedie “trim-and-fill” procedure [25]. Univariate meta-regression analyses were conducted to investigate associations between the effect size and the following parameters: age, proportion of males, sample size, and year of publication. Subgroup analyses were also conducted to investigate differences in effect size according to specific study design, clinical outcome studied, and country where the study was conducted.

The prognostic performance of the NLR was assessed using the Stata commands, metandi, midas, and mylabels. Summary receiver operating characteristic (SROC) curves were also generated using the hierarchical summary receiver operating characteristic (HSROC) model, complemented by empirical Bayes (EB) estimates, providing the best estimates of the true selectivity and specificity in each study [26,27]. Pooled sensitivity and specificity values, with corresponding forest plots, were calculated. The HSROC model also allows controlling for study heterogeneity, as determined by the (i) correlation coefficient between logit-transformed sensitivity and specificity (Corr(logits)) in HSROC analysis using a bivariate model [28] and (ii) asymmetry parameter β. A positive correlation coefficient (>0) and β value, with a *p*-value < 0.05, indicates the presence of heterogeneity [26,29]. Heterogeneity was further assessed through visual examination of the HSROC curve and using a bivariate boxplot (midas command). Publication bias was assessed using the Deeks’ method [30]. The relationships between pre-test probability, likelihood ratio, and post-test probability, were evaluated using the Fagan’s nomogram plot [31]. All analyses were performed using Stata 14 (StataCorp LLC, College Station, TX, USA).

## 3. Results

### 3.1. Study Selection

**Figure 1:**
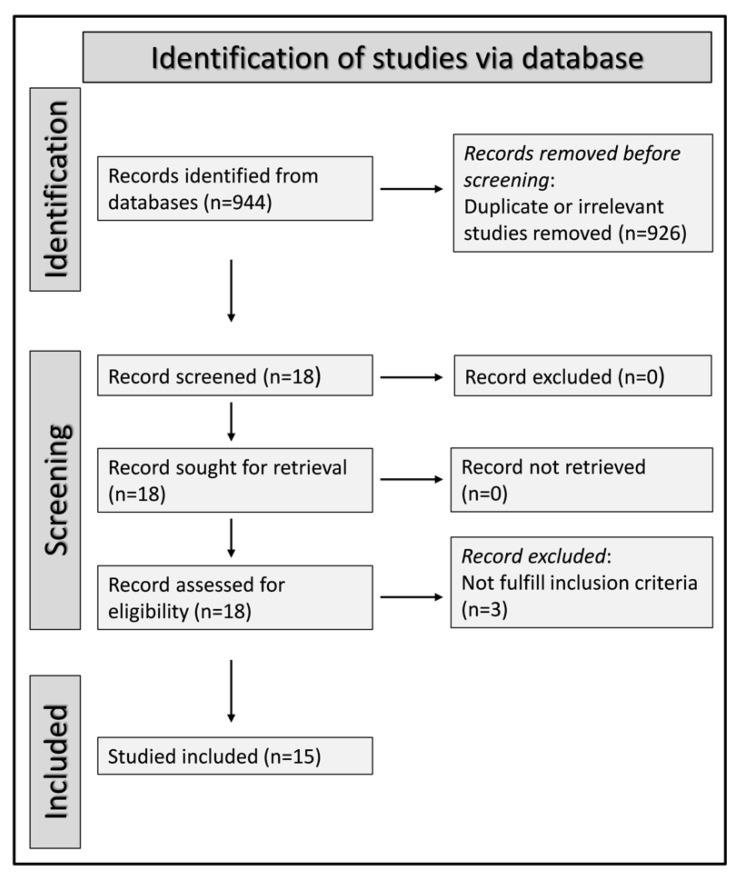
Preferred Reporting Items for Systematic reviews and Meta-Analyses (PRISMA) 2020 flow diagram.

We initially identified 944 articles. Of them, 926 were excluded because they were either duplicates or irrelevant. Following a full-text review of the remaining 18 articles, a further 3 were excluded because they did not fulfill the inclusion criteria, leaving 15 articles for final analysis (Figure 1 and Table 1) [32,33,34,35,36,37,38,39,40,41,42,43,44,45,46]. In all studies, the NLR was assessed within the first 24–48 h of admission.

### 3.2. Pooled Odds Ratios

#### 3.2.1. Study Characteristics

Fifteen studies (seventeen patient groups) of 10,038 AECOPD patients (57% males, mean age 74 years) reported associations between the NLR and adverse outcomes, expressed as ORs by multivariate logistic regression analysis. The adverse outcomes studied included mortality (nine studies) [33,34,35,36,37,38,40,43,45], transfer to ICU (one study) [36], length of hospital stay (one study) [46], invasive mechanical ventilation (IMV) (one study) [20], non-invasive mechanical ventilation failure (NIMVF) (one study) [44], pulmonary hypertension (one study) [39], and composite endpoints (two studies: ICU admission or death; one study: ICU admission, IMV, or death) [32,41,42]. Seven studies investigated in-hospital mortality [32,34,35,38,40,41,42], three 28-day mortality [36,43,45], and two 90-day mortality [33,37]. Eight studies were conducted in China [36,37,39,42,43,44,45,46], two in Iran [34,40], two in Turkey [35,38], one in Egypt [32], one in Australia [33], and one in Colombia [41] (Table 1).

#### 3.2.2. Risk of Bias

All studies had a low risk of bias according to the JBI checklist (Table 2).

#### 3.2.3. Results of Individual Studies and Syntheses

**Figure 2:**
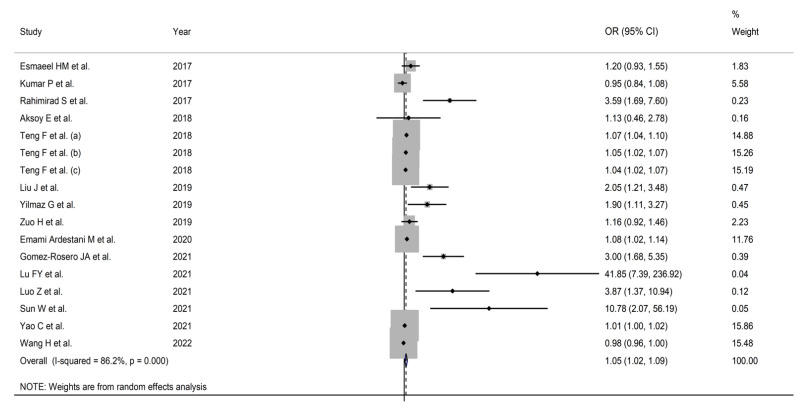
Forest plot of the association (odds ratio) between the neutrophil-to-lymphocyte ratio and adverse outcomes in AECOPD patients. *p* = 0.000 indicates a *p*-value < 0.001.

**Figure 3:**
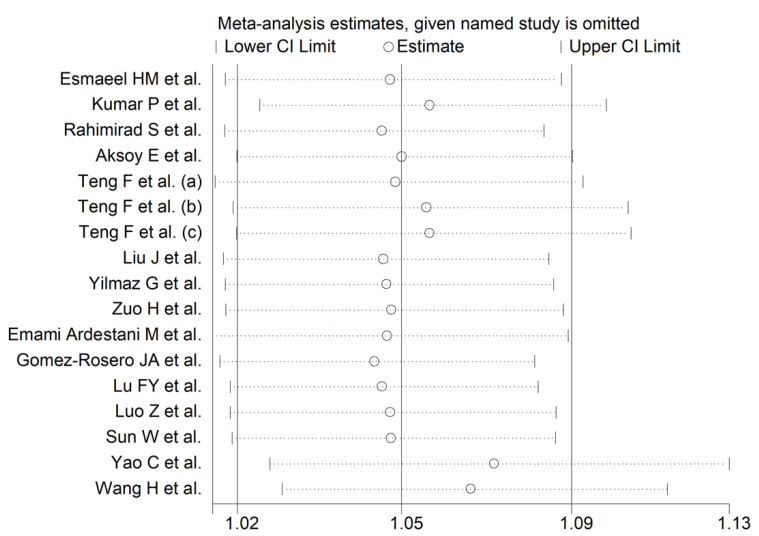
Sensitivity analysis showing the influence of individual studies on the overall odds ratio. The middle vertical axis indicates the overall odds ratio, and the two vertical axes indicate the 95% confidence intervals. The hollow circles represent the pooled odds ratio of the remaining studies when an individual study is omitted from the meta-analysis. The two ends of each broken line represent the 95% confidence intervals.

In twelve patient groups, higher NLR values were significantly associated with adverse outcomes [34,36,37,38,40,41,42,43,44,45]. By contrast, no significant associations were reported in five [32,33,35,39,46]. Pooled results showed that the NLR was significantly associated with adverse outcomes (OR = 1.054, 95% CI 1.016 to 1.093, *p* = 0.005) (Figure 2). The substantial between-study heterogeneity observed (I^2^ = 86.2%, *p* < 0.001) warranted the use of random-effects models. In sensitivity analysis, the corresponding pooled ORs were not substantially altered when individual studies were removed, suggesting that the results of the meta-analysis were stable (OR range, between 1.048 and 1.075) (Figure 3).

#### 3.2.4. Publication Bias

**Figure 4:**
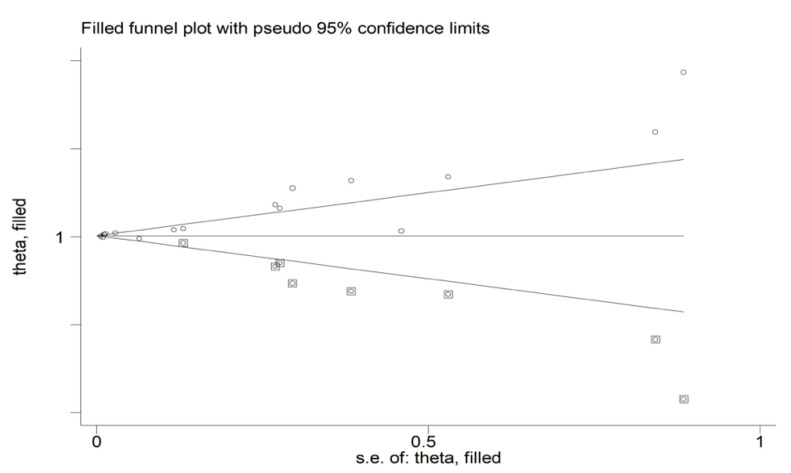
Funnel plot of studies investigating the association between the neutrophil-to-lymphocyte ratio and adverse outcomes after trimming and filling. Dummy studies and genuine studies are represented by enclosed circles and free circles, respectively.

There was evidence of publication bias according to the Begg’s (*p* = 0.020) and the Egger’s (*p* = 0.001) tests. The “trim-and-fill” method identified seven missing studies to be added to the left side of the funnel plot to ensure symmetry (Figure 4). However, the resulting effect size was not substantially different from the primary analysis (OR 1.032, 95% CI 0.988 to 1.078, *p* = 0.16).

#### 3.2.5. Subgroup and Meta-Regression Analysis

**Figure 5:**
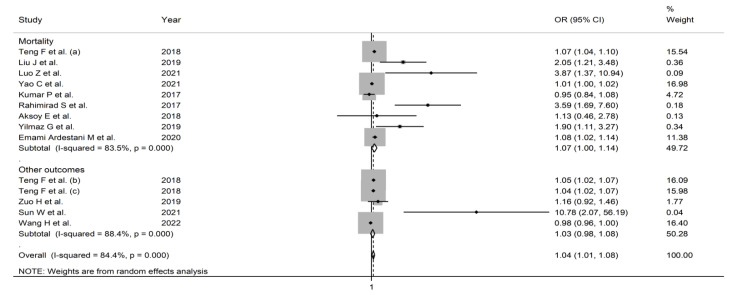
Forest plot of the association between the neutrophil-to-lymphocyte ratio, using odds ratios, and adverse outcomes according to specific endpoints (mortality vs. other outcomes). *p* = 0.000 indicates a *p*-value < 0.001.

**Figure 6:**
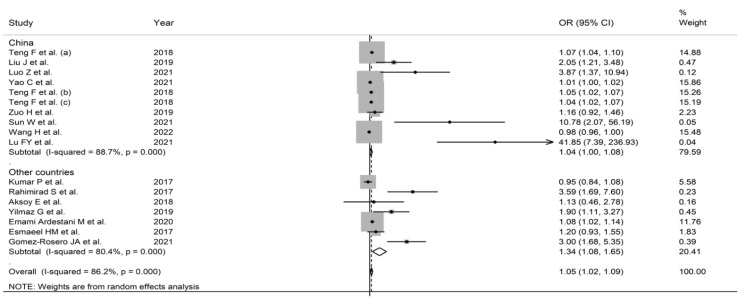
Forest plot of the association between the neutrophil-to-lymphocyte ratio, using odds ratios, and adverse outcomes according to the country where the study was performed (China vs. other countries). *p* = 0.000 indicates a *p*-value < 0.001.

**Figure 7:**
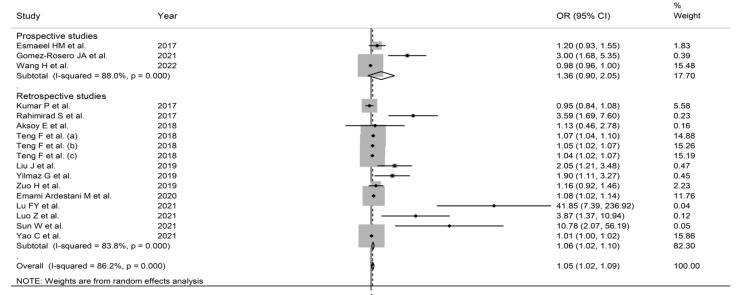
Forest plot of the association between the neutrophil-to-lymphocyte ratio, using odds ratios, and adverse outcomes according to study design (prospective vs. retrospective). *p* = 0.000 indicates a *p*-value < 0.001.

In subgroup analysis, the pooled OR in studies reporting mortality (1.067, 95% CI 1.002 to 1.136, *p* = 0.042; I^2^ = 83.5%, *p* < 0.001) was non-significantly different (*p* = 0.68) than that in studies reporting other adverse outcomes (1.029, 95% CI 0.982 to 1.079, *p* = 0.232; I^2^ = 88.4%, *p* < 0.001) (Figure 5). The pooled OR in studies conducted in China (1.039, 95% CI 1.002 to 1.0076, *p* = 0.039; I^2^ = 88.7%, *p* < 0.001) was non-significantly different (*p* = 0.56) than that in studies performed in other countries (1.336, 95% CI 1.080 to 1.652, *p* = 0.008; I^2^ = 80.4%, *p* < 0.001) (Figure 6). However, the pooled OR was significantly higher in retrospective studies (1.059, 95% CI 1.018 to 1.101, *p* = 0.004; I^2^ = 83.8%, *p* < 0.001) but not in prospective studies (1.359, 95% CI 0.903 to 2.047, *p* = 0.142; I^2^ = 88.0%, *p* < 0.001) (Figure 7). No significant associations were observed between the OR and age (t = 1.01, p = 0.36), proportion of males (t = 0.91, *p* = 0.41), sample size (t = −0.37, *p* = 0.73), or year of publication (t = 0.66, *p* = 0.54) in univariate meta-regression analysis.

#### 3.2.6. Certainty of Evidence

The initial level of certainty for NLR OR values was considered moderate because of the longitudinal nature of the selected studies (rating 3, ⊕⊕⊕⊝). After considering the low risk of bias in all studies (no rating change required), the substantial and unexplained heterogeneity (downgrade one level), the lack of indirectness (no rating change required), the relatively low imprecision (relatively narrow confidence intervals without threshold crossing, no rating change required), the small effect size (OR 1.054, no rating change required) [47], and the presence of publication bias, which was addressed with the “trim-and-fill” method (no rating change required), the overall level of certainty was downgraded to low (rating 2, ⊕⊕⊝⊝).

### 3.3. Pooled Standard Mean Differences

#### 3.3.1. Study Characteristics

Thirteen studies also reported absolute NLR values in 6,237 AECOPD patients (mean age 73 years, 51% males) without adverse outcomes and 787 AECOPD patients (mean age 75 years, 67% males) with adverse outcomes. Seven studies were conducted in China [37,39,42,43,44,45,46], two in Iran [34,40], two in Turkey [35,38], one in Australia [33], and one in Colombia [41]. The adverse outcomes assessed included mortality (eight studies) [33,34,35,37,38,40,43,45], length of hospital stay (one study) [46], NIMVF (one study) [44], pulmonary hypertension (one study) [39], and composite endpoints (one study: mortality or ICU admission; one study: ICU admission, IMV, or death) [41,42]. Six studies investigated in-hospital mortality [34,35,38,40,41,42], two 28-day mortality [43,45], and two 90-day mortality [33,37] (Table 3).

#### 3.3.2. Risk of Bias

The risk of bias was low in all studies (Table 2).

#### 3.3.3. Results of Individual Studies and Syntheses

**Figure 8:**
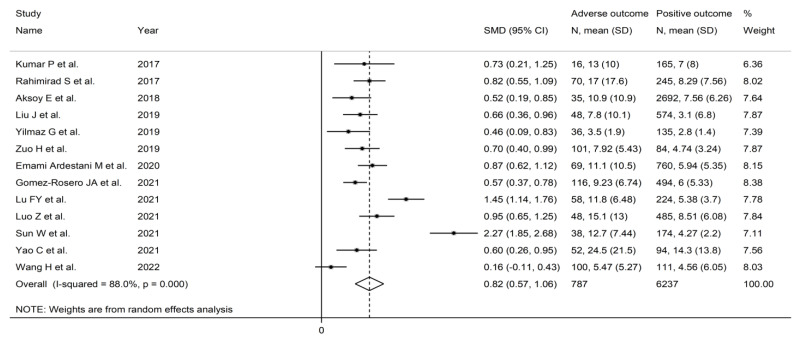
Forest plot of neutrophil-to-lymphocyte ratio values in AECOPD patients with and without adverse outcomes. *p* = 0.000 indicates a *p*-value < 0.001.

**Figure 9:**
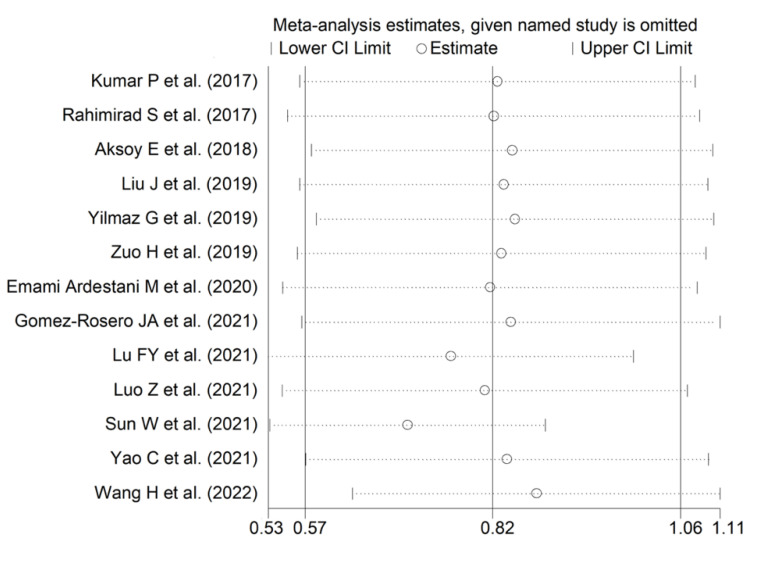
Sensitivity analysis of the association between the neutrophil-to-lymphocyte ratio and adverse outcomes, showing the influence of individual studies on the overall SMD. The middle vertical axis indicates the overall SMD, and the two vertical axes indicate the 95% confidence intervals. The hollow circles represent the pooled SMD of the remaining studies when an individual study is omitted from the meta-analysis. The two ends of each broken line represent the 95% confidence intervals.

The forest plot for NLR values in patients with and without adverse outcomes is shown in Figure 8. In all studies, patients with unfavourable outcomes had higher NLR values on admission than subjects without (mean difference range, 0.46 to 2.27), although the difference was not statistically significant in one study [46]. Substantial heterogeneity between studies was observed (I^2^ = 88.0%, *p* < 0.001). Thus, random-effects models were used. Overall, pooled results showed that NLR values were significantly higher in AECOPD patients experiencing adverse outcomes (SMD = 0.82, 95% CI 0.57 to 1.06, *p* < 0.001). Sensitivity analysis showed that the corresponding pooled SMD values were not substantially altered when individual studies was sequentially omitted (effect size range, between 0.71 and 0.88, Figure 9).

#### 3.3.4. Publication Bias

**Figure 10:**
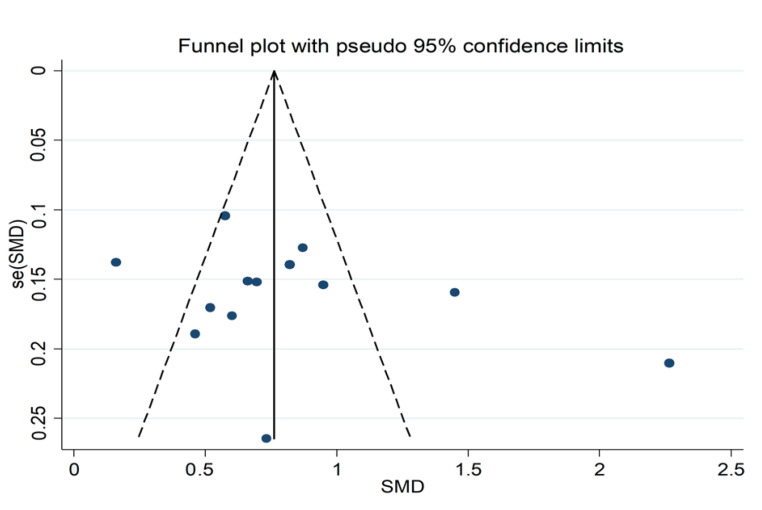
Funnel plot of studies investigating neutrophil-to-lymphocyte ratio values in AECOPD patients with and without adverse outcomes.

No publication bias was found either with the Begg’s (*p* = 0.43) or the Egger’s (*p* = 0.25) test. Accordingly, the “trim-and-fill” method did not identify any missing studies to be added to the funnel plot. However, a distortive effect of one study was observed [28] (Figure 10). Its removal did not significantly affect the effect size (SMD = 0.71, 95% CI 0.53 to 0.89, *p* < 0.001) or the heterogeneity (I^2^ = 76.6%, *p* < 0.001).

#### 3.3.5. Subgroup and Meta-Regression Analysis

**Figure 11:**
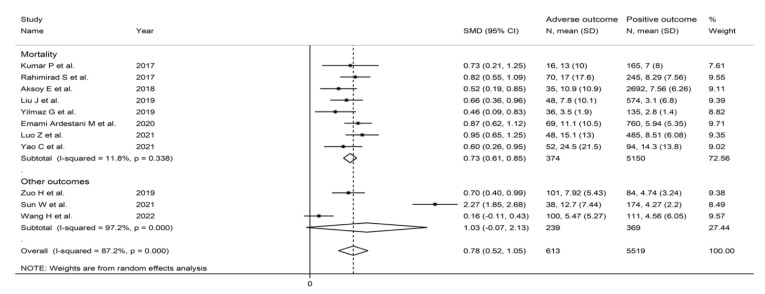
Forest plot of the association between the neutrophil-to-lymphocyte ratio, using standard mean differences, and adverse outcomes according to specific endpoints (mortality vs. other outcomes). *p* = 0.000 indicates a *p*-value < 0.001.

**Figure 12:**
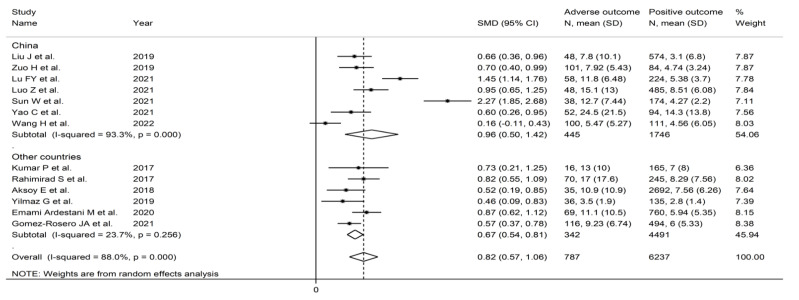
Forest plot of the association between the neutrophil-to-lymphocyte ratio, using standard mean differences, and adverse outcomes according to the country where the study was performed (China vs. other countries). *p* = 0.000 indicates a *p*-value < 0.001.

**Figure 13:**
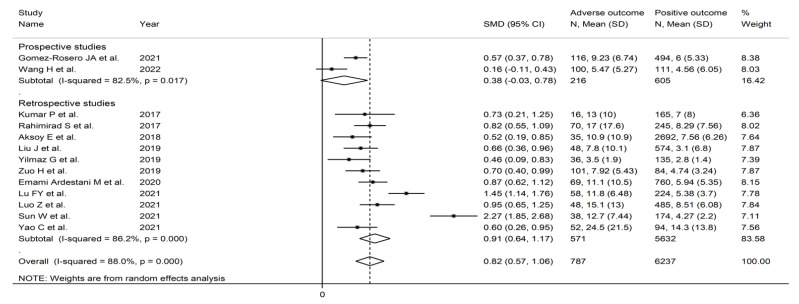
Forest plot of the association between the neutrophil-to-lymphocyte ratio, using standard mean differences, and adverse outcomes according to study design (prospective vs. retrospective). *p* = 0.000 indicates a *p*-value < 0.001.

In subgroup analysis, the SMD was significantly higher in studies investigating mortality (SMD = 0.73, 95% CI 0.61 to 0.85, *p* < 0.001; I^2^ = 11.8%; *p* = 0.338) but not in those assessing other adverse outcomes (SMD = 1.03, 95% CI −0.07 to 2.13, *p* = 0.07; I^2^ = 97.2%, *p* < 0.001) (Figure 11). The heterogeneity was also significantly lower in studies assessing mortality (I^2^ = 11.8% vs. I^2^ = 97.2%). There were no significant differences in effect size between studies performed in China (SMD = 0.96, 95% CI 0.50 to 0.42, *p* < 0.001; I^2^ = 93.3%; *p* < 0.001) and those performed in other countries (SMD = 0.67, 95% CI 0.54 to 0.81, *p* < 0.001; I^2^ = 23.7%; *p* = 0.356), although the heterogeneity was markedly higher in the former group (I^2^ = 93.3% vs. I^2^ = 23.8%) (Figure 12). However, the SMD was significantly higher in retrospective studies (SMD = 0.91, 95% CI 0.64 to 1.17, *p* < 0.001; I^2^ = 86.2%; *p* = 0.338) but not in prospective studies (SMD = 0.38, 95% CI−0.03 to 0.78, *p* = 0.07; I^2^ = 82.5%, *p* < 0.001) (Figure 13). No significant associations were observed between the SMD and age (t = 1.06, *p* = 0.31), proportion of males (t = 1.23, *p* = 0.25), sample size (t = −0.56, *p* = 0.59), or year of publication (t = 0.58, *p* = 0.57) in univariate meta-regression analysis.

#### 3.3.6. Certainty of Evidence

The initial level of certainty for NLR SMD values was considered moderate because the studies were longitudinal (rating 3, ⊕⊕⊕⊝). After further considering the low risk of bias in all studies (no rating change required), the substantial heterogeneity that was at least partly explained by the type of clinical outcome studied and the country where the study was conducted (no rating change required), the lack of indirectness (no rating change required), the relatively low imprecision (relatively narrow confidence intervals without threshold crossing, no rating change required), the large effect size (SMD = 0.82, upgrade one level) [48], and the absence of publication bias (no rating change required), the overall level of certainty was upgraded to high (rating 4, ⊕⊕⊕⊕).

### 3.4. Prognostic Accuracy of the NLR

#### 3.4.1. Study Characteristics

Eleven studies (thirteen treatment arms) in 6091 AECOPD patients (63% males, mean age 77 years) reported AUROC, sensitivity, specificity, and cut-off values to predict adverse outcomes [32,34,36,37,38,39,40,42,43,44,45]. Seven studies were conducted in China [36,37,39,42,43,44,45], two in Iran [34,40], one in Turkey [38], and one in Egypt [32]. Adverse outcomes studied included mortality (seven studies) [34,36,37,38,40,43,45], IMV (one study) [36], ICU admission (one study) [36], NIMVF (one study) [44], pulmonary hypertension (one study) [39], and composite endpoints (one study: mortality or ICU admission; one study: ICU admission, IMV, or death) [32,42]. Five studies investigated in-hospital mortality [32,34,38,40,42], three 28-day mortality [36,43,45], and one 90-day mortality [37] (Table 4).

#### 3.4.2. Risk of Bias

The risk of bias was low in all studies (Table 2).

#### 3.4.3. Results of Individual Studies and Syntheses

**Figure 14:**
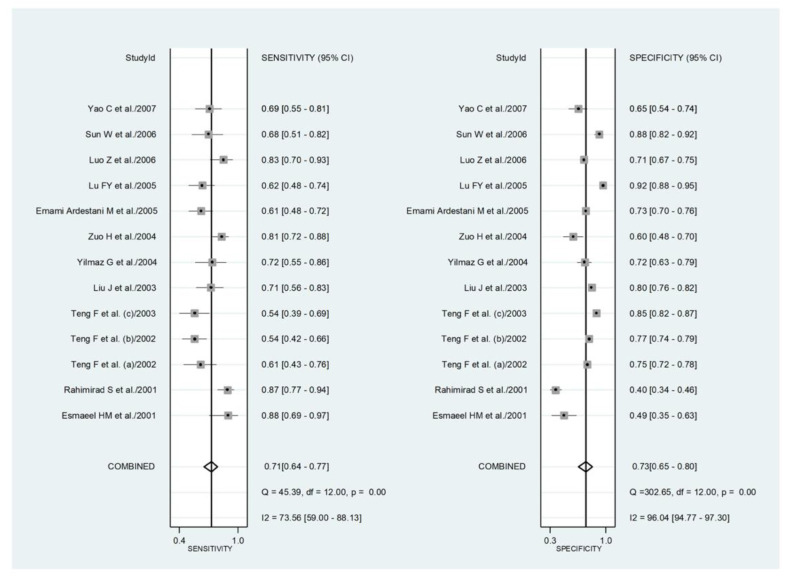
Forest plot for the pooled estimates of sensitivity and specificity of neutrophil-to-lymphocyte ratio values for predicting adverse outcomes. *p* = 0.00 indicates a *p*-value < 0.001.

**Figure 15:**
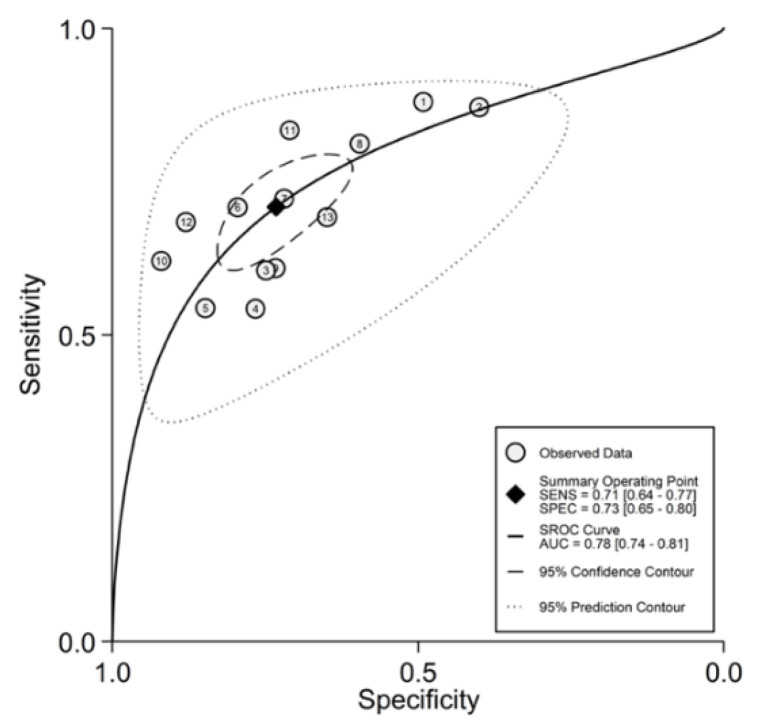
SROC curve with 95% confidence region and prediction region for neutrophil-to-lymphocyte ratio and adverse outcome prediction.

**Figure 16:**
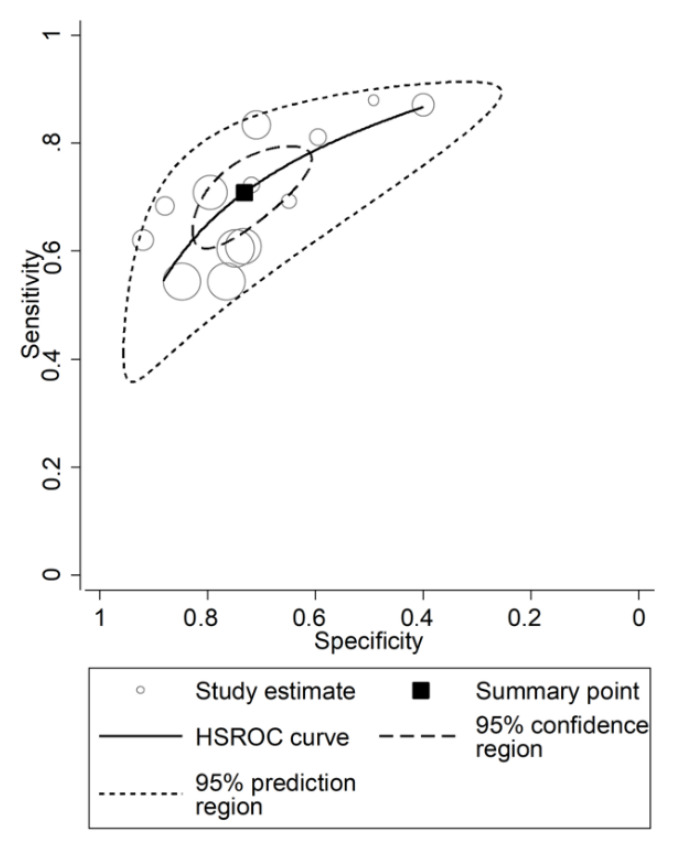
Empirical Bayes (posterior prediction) estimates of hierarchical receiving operating characteristic (HSROC) curve with 95% confidence region and prediction region for neutrophil-to-lymphocyte ratio in adverse outcome prediction.

**Figure 17:**
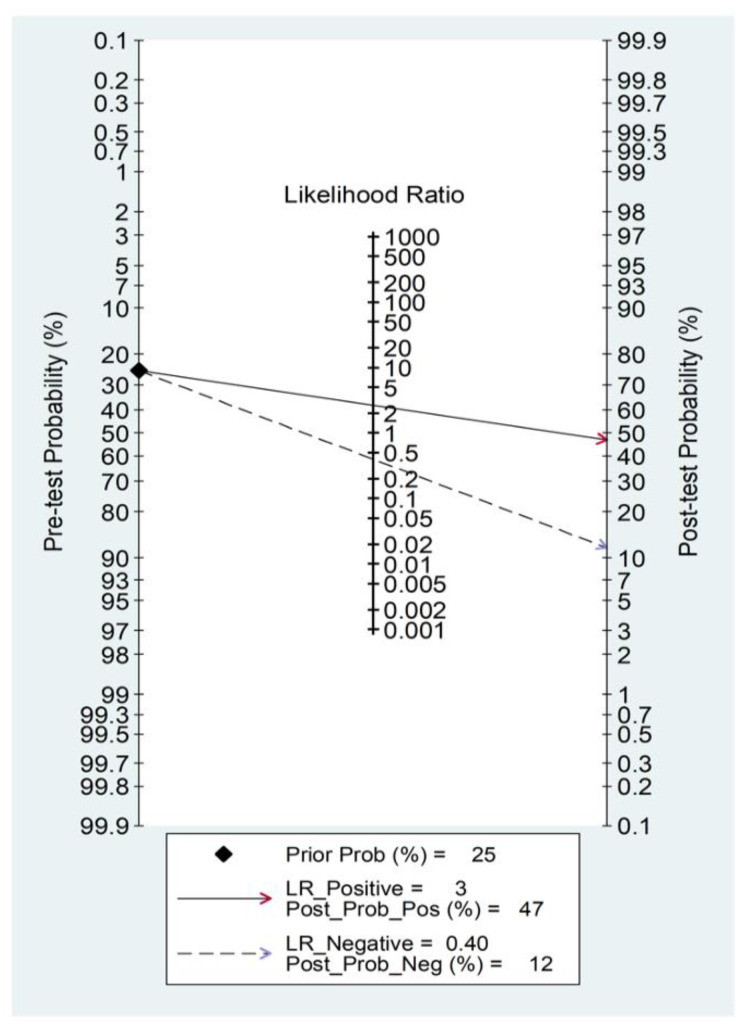
Fagan’s nomogram for the neutrophil-to-lymphocyte ratio in adverse outcome prediction.

Initially, forest plots for pooled sensitivity and specificity values were generated. Then, summary receiver operating characteristic (SROC) curves were generated using the HSROC model (midas or metandi command). The pooled sensitivity for NLR in predicting adverse outcomes was 0.71 (95% CI 0.64 to 0.77), and the pooled specificity was 0.73 (95% CI 0.65 to 0.80) (Figure 14). The SROC curve with 95% confidence region and prediction region is shown in Figure 15. The AUC value was 0.78 (95% CI 0.74 to 0.81), with the summary operating point set at sensitivity of 0.71 and specificity of 0.73. For the HSROC model (Figure 16), the pooled estimate and 95% CI of sensitivity and specificity were 0.71 (0.64 to 0.77) and 0.73 (0.65 to 0.80), respectively, which were identical to those obtained using the bivariate model. The Fagan’s nomogram (Figure 17), assuming a 25% incidence of overall adverse outcomes (pre-test probability), showed that the post-test probability of adverse outcome was 47% in patients with a relatively high NLR and 12% in those with a relatively low NLR.

#### 3.4.4. Publication Bias

**Figure 18:**
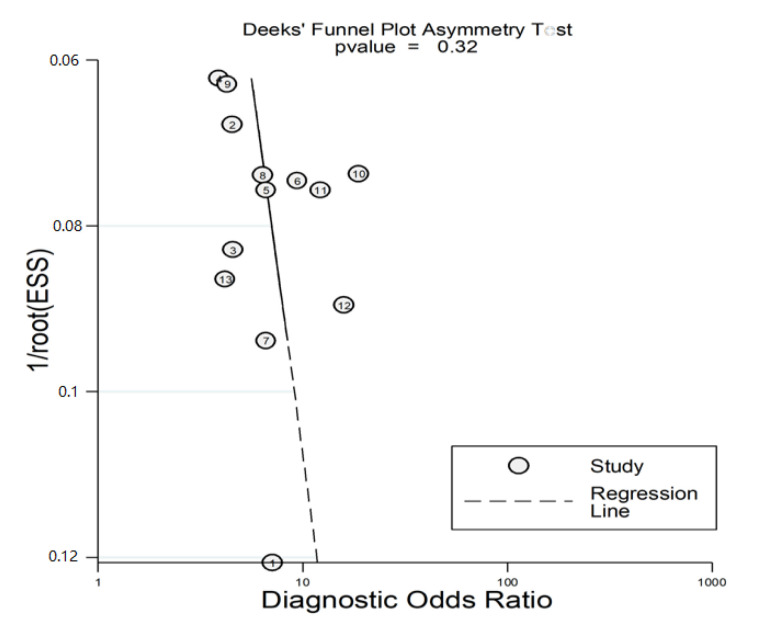
Deeks’ funnel plot asymmetry test for assessment of publication bias.

There was no significant publication bias using the Deeks’ funnel plot asymmetry test (the scatter plot was symmetrical, and the *p*-value was *>* 0.05) (Figure 18).

#### 3.4.5. Heterogeneity, Subgroup and Meta-Regression Analysis

**Figure 19:**
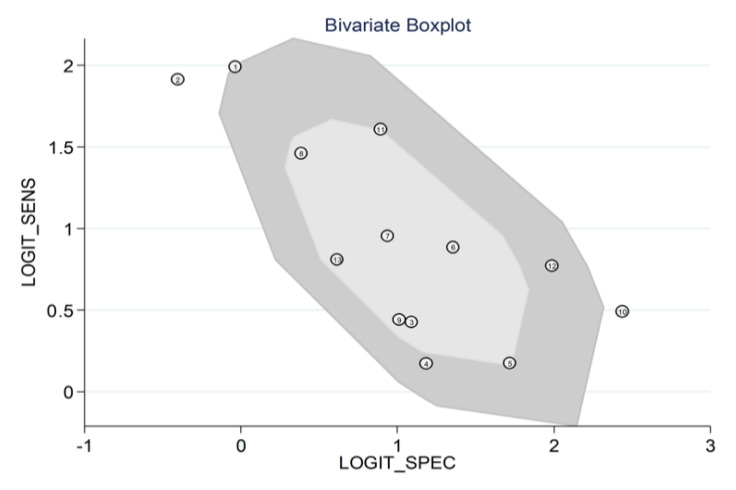
Bivariate boxplot exploring heterogeneity across studies.

**Figure 20:**
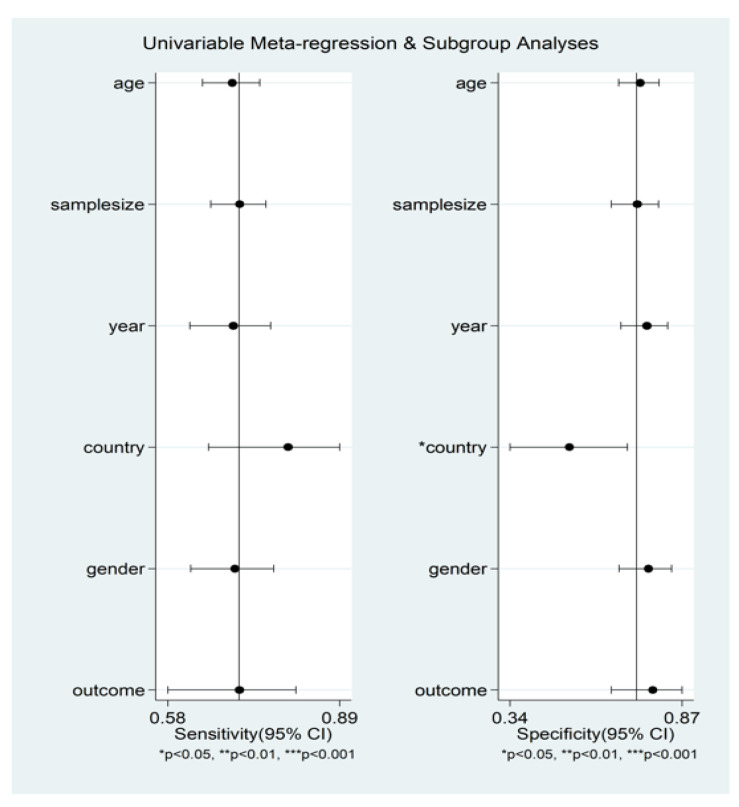
Forest plots of sensitivity and specificity for the study level covariates included in the univariate meta-regression model.

Heterogeneity was assessed using different methods. First, the HSROC curve (Figure 19) was shown to be symmetric, based on (i) the negative correlation coefficient between logit-transformed sensitivity and specificity (−0.856, 95% CI −0.978 to −0.289) and (ii) the non-significant symmetry parameter β (0.355, 95% CI −0.155 to 0.866, *p* = 0.171). This indicates no heterogeneity between studies [26,29]. However, the visual representation of the HSROC curve (Figure 16) suggests a moderate degree of heterogeneity (95% CI 0.74 to 0.81). Using the midas command, pooled sensitivity and specificity showed an inconsistency (I^2^) of 73.56% and 96.04%, respectively (Figure 14). The bivariate boxplot using the logit_Se and logit_Sp commands (Figure 19) showed that two studies [34,42] fell outside the circles, indicating the presence of heterogeneity. The sources of the observed heterogeneity were further explored using univariate meta-regression analysis. As reported in Figure 20, the country where the study was conducted was significantly associated with specificity (*p* = 0.03). This significantly decreased the specificity to 0.52 (95% CI 0.34 to 0.70), without affecting the sensitivity (*p* = 0.24).

## 4. Discussion

In our systematic review and meta-analysis, the NLR measured within 24–48 h of hospital admission was significantly associated, both in terms of ORs and SMDs, with the risk of several adverse events during hospitalization and, particularly, short-term mortality up to 90 days in AECOPD. In meta-regression, we did not observe any significant association between the effect size and pre-defined patient and study characteristics. However, in subgroup analysis, the SMD was significantly higher in studies investigating mortality compared to those assessing other adverse outcomes. Assessment of the predictive capacity of the NLR towards adverse outcomes, using the HSROC model, yielded an AUC value of 0.78, which indicates good prognostic accuracy [49,50].

The NLR, a cell inflammatory index that is easily derived from routine haematological parameters, is increasingly being studied as a diagnostic and prognostic biomarker in a wide range of disease states [51,52,53,54,55,56,57,58]. In the context of COPD, several observational studies have reported that, in the stable phase of the disease, the NLR is significantly higher in patients with specific comorbidities, e.g., lung cancer and metabolic syndrome, and those at risk of AECOPD [16,17]. These observations support the proposition that the presence of relatively higher NLR values reflects a local (airways) and/or systemic pro-inflammatory state. However, relatively little has been known regarding the prognostic capacity of the NLR specifically in patients experiencing AECOPD, as studies addressing this issue have only been published since 2017 [32,33,34,35,36,37,38,39,40,41,42,43,44,45,46]. Whilst the articles identified in our systematic review investigated a range of adverse outcomes during hospitalization (e.g., ICU admission, need for mechanical ventilation, length of stay, and pulmonary hypertension), the most studied outcome was short-term mortality up to 90 days from admission. This issue notwithstanding, the NLR was able to significantly discriminate between AECOPD patients with and without adverse outcomes regardless of whether we analysed ORs or SMDs. The good prognostic accuracy of the NLR, based on the pooled AUC value of 0.78, further supports the potential prognostic role of this biomarker in AECOPD. However, further research is warranted to determine whether the NLR on admission should be routinely used, singly or in combination with other biomarkers or clinical characteristics, to guide personalized clinical decisions regarding management. The different capacity of single vs. combined biomarkers to predict outcomes has been previously highlighted in studies of AECOPD patients. In one study, individual circulating inflammatory biomarkers, e.g., C-reactive protein, cytokines, and white blood cell differentiation, were not superior to a history of previous AECOPD in predicting outcomes [59,60]. However, in other studies, the combination of C-reactive protein, neutrophil count, and presence of laboured breathing successfully discriminated between AECOPD and stable COPD [61]. Large, appropriately designed prospective studies should investigate the capacity of the NLR, other inflammatory biomarkers, and clinical characteristics to predict various adverse clinical outcomes. Such studies should investigate AECOPD patients with different aetiologies, capture longer follow-up periods, include serial NLR assessments and additional endpoints, e.g., risk of hospital readmission, and assess the effects of specific therapies, e.g., corticosteroids and anti-infective agents or their combinations.

Strengths of our study include the combined meta-analytical assessment of ORs through multivariate analysis, SMDs, and predictive performance of the NLR through the calculation of pooled AUC, sensitivity, and specificity. In addition, we conducted meta-regression and subgroup analyses to investigate associations between the effect size and specific study and patient characteristics and assessed the certainty of evidence using GRADE. One important limitation is the lack of studies conducted in European and North American cohorts, which limits the generalizability of our findings. In this context, an epidemiological study conducted in the USA has reported that the NLR was significantly lower in black participants than white participants, with intermediate values in Hispanics and other ethnic groups [62]. Additional studies are warranted to determine whether the association between the NLR and adverse outcomes in AECOPD is influenced by ethnicity. Another important limitation is represented by the substantial between-study heterogeneity in our analyses of ORs and SMDs. However, it is also important to emphasise that, in sensitivity analysis, the effect size was not substantially affected when individual studies were, in turn, removed. Furthermore, the conclusions need to be interpreted with caution given that only three of the fifteen studies identified were prospective. Finally, the lack of data provided in the articles identified regarding temporal changes in the NLR following admission does not allow us to establish the possible influence of specific pharmacological and non-pharmacological treatments on the studied outcomes.

## 5. Conclusions

Our systematic review and meta-analysis have shown that the NLR on admission is significantly associated, both in terms of ORs and as SMDs, with the risk of several adverse events during hospitalization, particularly short-term mortality up to 90 days, in AECOPD. Further prospective studies investigating other biomarkers over longer follow-up periods are warranted to establish the potential clinical use of the NLR, singly or as part of a combined predictive model, in early risk stratification and therapeutic decisions in patients with AECOPD.

## Figures and Tables

**Table 1:** Study characteristics.

First Author, Year, Country [Ref]	Study Design	Sample Size	OR (95% CI)	AUC (95% CI)	Cut-Off	Sensitivity (%)	Specificity (%)	Clinical Outcome
Esmaeel H.M., 2017, Egypt [32]	P	80	1.2 (0.9–1.5)	0.642 (0.526–0.746)	3.4	0.89	0.49	In-hospital mortality or ICU transfer
Kumar P., 2017, Australia [33]	R	181	0.95 (0.84–1.08)	NR	NR	NR	NR	90-day mortality
Rahimirad S., 2017, Iran [34]	R	174	3.586 (1.69–7.60)	0.717 (0.623–0.811)	4	0.87	0.4	In-hospital mortality
Aksoy E., 2018, Turkey [35]	R	2727	1.13 (0.46–2.78)	NR	NR	NR	NR	In-hospital mortality
Teng F. (a), 2018, China [36]	R	904	1.067 (1.039–1.095)	0.737 (0.661–0.814)	8.13	0.61	0.75	28-day mortality
Teng F. (b), 2018, China [36]	R	906	1.046 (1.023–1.068)	0.676 (0.607–0.744)	8.13	0.54	0.77	ICU transfer
Teng F. (c), 2018, China [36]	R	906	1.042 (1.019–1.066)	0.732 (0.656–0.807)	10.345	0.54	0.85	IMV
Liu J., 2019, China [37]	R	622	2.05 (1.21–3.48)	0.742 (0.554–0.881)	4.19	0.71	0.74	90-day mortality
Yilmaz G., 2019, Turkey [38]	R	171	1.902 (1.108–3.266)	NR	3.18	0.71	0.72	In-hospital mortality
Zuo H., 2019, China [39]	R	185	1.161 (0.924–1.458)	0.701 (0.629–0.766)	4.659	0.81	0.6	In-hospital PH
Emami Ardestani M., 2020, Iran [40]	R	829	1.08 (1.02–1.14)	0.7 (0.67–0.73)	6.9	0.61	0.73	In-hospital mortality
Gomez-Rosero J.A., 2021, Colombia [41]	P	610	3.0 (1.7–5.4)	NR	NR	NR	NR	In-hospital mortality or ICU transfer
Lu F.Y., 2021, China [42]	R	282	41.85 (9.57–306.74)	0.883 (0.771–0.894)	10.23	0.62	0.92	In-hospital mortality, ICU transfer, or IMV
Luo Z., 2021, China [43]	R	533	3.87 (1.29–10.3)	0.801 (NR)	6.74	0.83	0.71	28-day mortality
Sun W., 2021, China [44]	R	212	10.783 (2.069–56.194)	0.858 (0.785–0.931)	8.9	0.69	0.88	NIMVF
Yao C., 2021, China [45]	R	146	1.01 (0.999–1.022)	0.83 (0.761–0.899)	16.83	0.69	0.65	28-day mortality
Wang H., 2022, China [46]	P	598	0.98118 (0.96271–0.999)	NR	NR	NR	NR	LHS

Legend: OR, odds ratio; AUC, area under the curve; ICU, intensive care unit; IMV, invasive mechanical ventilation; LHS, length of hospital stay; NIMVF, non-invasive mechanical ventilation failure; NR, not reported; P, prospective; PH, pulmonary hypertension; R, retrospective.

**Table 2:** The Joanna Briggs Institute Critical Appraisal Checklist.

Study	Were the Groups Comparable Other than the NLR?	Were Cases and Controls Matched Appropriately?	Were the Same Criteria Used to Identify Cases and Controls?	Was Exposure Measured in a Standard, Valid, and Reliable Way?	Was Exposure Measured in the Same Way for Cases and Controls?	Were Confounding Factors Identified?	Were Strategies to Deal with Confounding Factors Stated?	Were Outcomes Assessed in a Standard, Valid, and Reliable Way for Cases and Controls?	Was the Exposure Period Long Enough to Be Meaningful?	Was Appropriate Statistical Analysis Used?	Risk of Bias
Esmaeel H.M. [32]	Yes	Yes	Yes	Yes	Yes	Yes	Yes	Yes	Yes	Yes	Low
Kumar P. [33]	Yes	Yes	Yes	Yes	Yes	Yes	Yes	Yes	Yes	Yes	Low
Rahimirad S. [34]	No	No	Yes	Yes	Yes	Yes	Yes	Yes	Yes	Yes	Low
Aksoy E. [35]	No	No	Yes	Yes	Yes	Yes	Yes	Yes	Yes	Yes	Low
Teng F. [36]	Yes	Yes	Yes	Yes	Yes	Yes	Yes	Yes	Yes	Yes	Low
Liu J. [37]	Yes	Yes	Yes	Yes	Yes	Yes	Yes	Yes	Yes	Yes	Low
Yilmaz G. [38]	Yes	Yes	Yes	Yes	Yes	Yes	Yes	Yes	Yes	Yes	Low
Zuo H. [39]	Yes	Yes	Yes	Yes	Yes	Yes	Yes	Yes	Yes	Yes	Low
Emami Ardestani M. [40]	No	No	Yes	Yes	Yes	Yes	Yes	Yes	Yes	Yes	Low
Gomez-Rosero J.A. [41]	Yes	Yes	Yes	Yes	Yes	Yes	Yes	Yes	Yes	Yes	Low
Lu F.Y. [42]	Yes	Yes	Yes	Yes	Yes	Yes	Yes	Yes	Yes	Yes	Low
Luo Z. [43]	Yes	Yes	Yes	Yes	Yes	Yes	Yes	Yes	Yes	Yes	Low
Sun W. [44]	Yes	Yes	Yes	Yes	Yes	Yes	Yes	Yes	Yes	Yes	Low
Yao C. [45]	Yes	Yes	Yes	Yes	Yes	Yes	Yes	Yes	Yes	Yes	Low
Wang H. [46]	Yes	Yes	Yes	Yes	Yes	Yes	Yes	Yes	Yes	Yes	Low

**Table 3:** Characteristics of studies reporting absolute neutrophil-to-lymphocyte values in AECOPD with and without adverse outcomes.

	Without Adverse Outcome				With Adverse Outcome				Outcome
First Author, Year, Country [Ref]	n	Age (Years)	Gender (M/F)	NLR (Mean ± SD)	n	Age (Years)	Gender (M/F)	NLR (Mean ± SD)	
Kumar P., 2017, Australia [33]	165	70	81/84	7 ± 8	16	78	12/4	13 ± 10	90-day mortality
Rahimirad S., 2017, Iran [34]	245	69	127/118	8.29 ± 7.56	70	74	47/23	17 ± 17.56	In-hospital mortality
Aksoy E., 2018, Turkey [35]	2,692	NR	1144/1548	7.56 ± 6.26	35	NR	23/12	10.85 ± 10.92	In-hospital mortality
Liu J., 2019, China [37]	574	74	281/293	3.1 ± 6.8	48	75	26/22	7.8 ± 10.1	90-day mortality
Yilmaz G., 2019, Turkey [38]	135	71	73/62	2.8 ± 1.4	36	69	23/13	3.5 ± 1.9	In-hospital mortality
Zuo H., 2019, China [39]	84	70	64/20	4.74 ± 3.24	101	72	77/34	7.92 ± 5.43	In-hospital PH
Emami Ardestani M., 2020, Iran [40]	760	68	502/258	5.94 ± 5.35	69	72	53/16	11.12 ± 10.51	In-hospital mortality
Gomez-Rosero J.A., 2021, Colombia [41]	494	75	233/261	6 ± 5.33	116	71	58/58	9.23 ± 6.74	In-hospital mortality or ICU transfer
Lu F.Y., 2021, China [42]	224	NR	NR	5.38 ± 3.7	58	NR	NR	11.77 ± 6.48	In-hospital mortality, ICU transfer, or IMV
Luo Z., 2021, China [43]	485	75	325/160	8.51 ± 6.08	48	81	30/18	15.12 ± 12.99	28-day mortality
Sun W., 2021, China [44]	174	73	123/51	4.27 ± 2.2	38	77	30/8	12.67 ± 7.44	NIMVF
Yao C., 2021, China [45]	94	78	67/27	14.3 ± 13.78	52	81	42/10	24.47 ± 21.48	28-day mortality
Wang H., 2021, China [46]	111	70	NR	4.56 ± 6.05	100	78	NR	5.47 ± 5.27	LHS

Legend: ICU, intensive care unit; IMV, invasive mechanical ventilation; LHS, length of hospital stay; NIMVF, non-invasive mechanical ventilation failure; NR, not reported; PH: pulmonary hypertension.

**Table 4:** Characteristics of studies reporting AUROC, sensitivity, specificity, and cut-off values for the neutrophil-to-lymphocyte ratio to predict adverse outcomes.

First Author, Year, Country [Ref]	n	Age (Years)	Gender (M/F)	AUC	95% CI	Sensitivity	Specificity	Cut-Off	Outcome
Esmaeel H.M., 2017, Egypt [32]	80	61	NR	0.642	0.526–0.746	0.8889	0.4906	3.4	ICU transfer or in-hospital mortality
Rahimirad S., 2017, Iran [34]	315	70	245/70	0.717	0.623–0.811	0.87	0.4	4	In-hospital mortality
Teng F. (a), 2018, China [36]	904	82	525/379	0.737	0.661–0.814	0.605	0.748	8.13	28-day mortality
Teng F. (b), 2018, China [36]	906	82	525/381	0.676	0.607–0.744	0.543	0.766	8.13	ICU transfer
Teng F. (c), 2019, China [36]	906	82	525/381	0.732	0.656–0.807	0.543	0.848	10.345	IMV
Liu J., 2019, China [37]	622	74	307/315	0.742	0.554–0.881	0.714	0.742	4.19	90-day mortality
Yilmaz G., 2019, Turkey [38]	171	71	96/75	NR	NR	0.71	0.72	3.18	In-hospital mortality
Zuo H., 2019, China [39]	185	71	141/54	0.701	0.629–0.766	0.812	0.595	4.659	In-hospital PH
Emami Ardestani M., 2020, Iran [40]	829	68	555/274	0.70	0.67–0.73	0.6087	0.7329	6.9	In-hospital mortality
Lu F.Y., 2021, China [42]	282	78	247/35	0.883	0.771–0.894	0.62	0.92	10.23	IMV, ICU transfer, or in-hospital mortality
Luo Z., 2021, China [43]	533	76	355/178	0.801	NR	0.83	0.71	6.74	28-day mortality
Sun W., 2021, China [44]	212	74	153/59	0.858	0.785–0.931	0.69	0.88	8.9	NIMVF
Yao C., 2021, China [45]	146	79	109/37	0.83	0.761–0.899	0.69	0.65	16.83	28-day mortality

Legend: ICU, intensive care unit; IMV, invasive mechanical ventilation; NIMVF, non-invasive mechanical ventilation failure; NR, not reported; PH: pulmonary hypertension.

## Data Availability

The data that support the findings of this systematic review and meta-analysis are available from the first author, A.Z., upon reasonable request.

## Supplementary Materials

The following supporting information can be downloaded at: https://www.mdpi.com/article/10.3390/jcm11123365/s1, Table S1: PRISMA 2020 abstract checklist; Table S2: PRISMA 2020 manuscript checklist.
